# Serological Investigation for *Brucella ceti* in Cetaceans from the Northwestern Mediterranean Sea

**DOI:** 10.3390/ani14162417

**Published:** 2024-08-20

**Authors:** Laura Martino, María Cuvertoret-Sanz, Sarah Wilkinson, Alberto Allepuz, Albert Perlas, Llilianne Ganges, Lola Pérez, Mariano Domingo

**Affiliations:** 1 Departament de Sanitat i Anatomia Animals, Facultat de Veterinària, Universitat Autònoma de Barcelona, 08193 Bellaterra, Spain; alberto.allepuz@uab.cat (A.A.); mariano.domingo@uab.cat (M.D.); 2 Servei de Diagnòstic de Patologia Veterinària, Facultat de Veterinària, Universitat Autònoma de Barcelona, 08193 Bellaterra, Spain; maria-cuvertoret@idexx.com (M.C.-S.); sarah@vetscape.co.za (S.W.); 3 IRTA—Institut de Recerca en Sanitat Animal, Programa de Sanitat Animal, Centre de Recerca en Sanitat Animal (CReSA), 08193 Bellaterra, Spain; llilianne.ganges@irta.cat; 4 Facultat de Veterinària de Barcelona, Universitat Autònoma de Barcelona, 08193 Bellaterra, Spain; lola.perez@uab.cat

**Keywords:** brucellosis, serology, antibodies, ELISA, morbillivirus, dolphin, stranding, necropsy

## Abstract

**Simple Summary:**

*Brucella ceti* is a marine bacterium that causes neurological, reproductive and skeletal disease in free-ranging cetaceans. Its zoonotic potential and importance for wild animals has prompted, over the years, the search for a reliable diagnostic method to detect antibodies and infer the level of infection. In this work, we perform an exploratory serological study on cetaceans stranded in the Northwestern Mediterranean Sea. Antibody levels were higher in animals with confirmed *Brucella* disease and infection in juveniles and in animals with chronic morbilliviral infection. This provides the first seroprevalence estimation in this area and reaffirms the active circulation of *Brucella* in wild cetaceans.

**Abstract:**

Neurobrucellosis in cetaceans, caused by *Brucella ceti*, is a relevant cause of death in striped dolphins (*Stenella coeruleoalba*) from the Mediterranean Sea. Serological tests are not used as a routinary technique for the diagnosis of this infection. We briefly describe the pathological findings of nine free-ranging stranded cetaceans diagnosed with *Brucella* disease or infection in our veterinary necropsy service from 2012 to 2022. The findings included focal diskospondylitis and non-suppurative meningitis, choroiditis and radiculitis. Additionally, an exploratory serological study was conducted in sixty-six frozen sera collected in the period 2012–2022 from fifty-seven striped dolphins, five Risso’s dolphins (*Grampus griseus*), two common bottlenose dolphins (*Tursiops truncatus*), one common dolphin (*Delphinus delphis*) and one pilot whale (*Globicephala melas*) to compare antibody levels in *Brucella*-infected (n = 8) and non-infected (n = 58) animals, classified by the cause of death, sex, age class and cetacean morbillivirus (CeMV) infection status. The authors hypothesized that active infection in cases of neurobrucellosis would elicit a stronger, detectable humoral response compared to subclinical infections. We performed a commercial competition ELISA (cELISA) using serial serum dilutions for each sample, considering a percentage of inhibition (PI) of ≥40% as positive. A titer of 1:160 was arbitrarily determined as the seropositivity threshold. Seropositive species included striped dolphins and Risso’s dolphins. Seroprevalence was higher in animals with neurobrucellosis (87.5%) compared to the overall seroprevalence (31.8%) and to other causes of death, indicating, likely, a high sensitivity but low specificity for neurobrucellosis. Animals with chronic CeMV seemed to have higher seroprevalences, as well as juveniles, which also had a higher disease prevalence. These results indicate, as in other studies, that antibodies are not decisive against clinical brucellosis, although they may indicate a carrier state, and that CeMV may influence *Brucella* epidemiology. More research is required to elucidate the epidemiology and pathogenesis and to resolve the complicated host–pathogen interaction in *Brucella* species.

## 1. Introduction

Brucellosis in cetaceans was first described in 1994 [1] and is caused by *Brucella ceti* [2]. Since then, infection and disease associated with *B. ceti* have been increasingly recognized in many cetacean species worldwide [3,4], and brucellosis is considered an emerging threat for odontocetes and mysticetes [5]. In the Mediterranean Sea, cetacean brucellosis was first diagnosed in 2013, and more cases have been reported since then [6,7,8,9,10], but serological evidence of exposure was already detected in dolphins stranded along the Mediterranean coast of Spain in 1997–1999 [11], suggesting that Mediterranean dolphins were already exposed to the pathogen at that time. Disease due to *B. ceti* in cetaceans can occur as neurobrucellosis, reproductive disease, spinal diskospondylitis or abscesses (frequently cutaneous and subcutaneous) [4]. However, in many other instances, serological evidence of infection has been found in asymptomatic cetaceans, and *B. ceti* has been isolated from tissues of apparently healthy cetaceans, suggesting that *Brucella*-infected cetaceans may overcome initial infection and survive or become *Brucella* carriers (see for review [3,4]). Health assessment of wild cetacean populations is usually conducted through necropsy of stranded dolphins and laboratory investigation. Since the first description of disease associated with *B. ceti* in the Catalan Mediterranean coast [8], we have intensified efforts to detect *Brucella* in stranded cetaceans. In this paper we describe the cases of brucellosis detected in the period 2012 to 2022 in the Northwestern Mediterranean coast. Moreover, we perform an exploratory serological analysis using a commercial cELISA to compare the antibody levels between infected and non-infected animals to determine if animals with *Brucella*-associated lesions have higher antibody titers than potential subclinical carriers. Serological results were compared by age groups, sex, and cetacean morbillivirus (CeMV) status, with a special emphasis on striped dolphins. CeMV is assessed routinely in all cetaceans, and in *Brucella* cases, the coinfections were investigated because of the reports of coinfection in other areas [12] and to explore the effect CeMV infection has on anti-*Brucella* antibody production. The general aims were to provide information about the epidemiology and pathogenesis of *B. ceti* infection and to evaluate its impact on cetacean species in the area.

## 2. Materials and Methods

### 2.1. Cases Investigated and Necropsy Procedure

A total of 123 cetaceans stranded along the Catalan coast during the period 2012–2022 were necropsied at the Universitat Autònoma de Barcelona, according to standard procedures [13]. Necropsied species included striped dolphins (*Stenella coeruleoalba*) (n = 97), common bottlenose dolphins (*Tursiops truncatus*) (n = 11), Risso’s dolphins (*Grampus griseus*) (n = 10), common dolphins (*Delphinus delphis*) (n = 2), a fin whale (*Balaenoptera physalus*), a pilot whale (*Globicephala melas*) and a Cuvier’s beaked whale (*Ziphius cavirostris*). Condition code ranged from 1 to 3 (animals in overt autolysis were not transported for necropsy) following the scale of Kuiken and García-Hartmann 1991 [14]. Retrieval of cerebrospinal fluid (CSF) was routinely attempted from the atlanto-occipital joint, or directly from the lateral ventricle after extraction of the brain. Frozen samples and swabs from multiple organs were collected for bacteriological and molecular investigations. A complete set of organs was sampled for histopathology, fixed in 10% neutral buffered formalin and routinely processed. Routine immunohistochemistry and RT-PCR for surveillance of CeMV were performed on lung, diaphragmatic lymph node and brain [15]. Cause of death was determined using a combination of computed tomography scan, complete necropsy, routine histopathology of multiple organs, CeMV PCR and immunohistochemistry (IHC) and, when required, microbiological culture (see [15] for details). After the determination of the cause of death, animals were classified in different groups: bycatch, neurobrucellosis, CeMV, PEM (polioencephalomalacia of unknown origin), mother–calf separation, infectious/inflammatory causes, sinusitis by *Crassicauda grampicola*, other and unknown. “Infectious/inflammatory” cause of death comprised peritonitis, septicemia, protozoal infections, severe parasitism (other than *C. grampicola*), diskospondylitis, mucormycosis, bacterial meningoencephalitis (negative *Brucella* culture), necrotizing enteritis, erysipelas and meningoencephalitis of unknown origin. “Other” included animals dying from tension pneumothorax, abortion and muscular degeneration.

### 2.2. Serology for Brucella

Blood retrieval was attempted by cardiac puncture as soon as possible after reception of each cadaver with a 20 mL syringe and a 16G, 1.7 × 133 mm catheter needle (Angiocath^™^, BD, REF 382259, Franklin Lakes, NJ, USA) and portioned in vacutainer plain tubes. Blood was centrifuged at 2500× *g* for 10 min, and serum was aliquoted in 1 mL cryotubes and frozen at −80 °C until use.

For the present serological study, available serum samples from 2012 to 2022 (n = 66; 57 striped dolphins, 5 Risso’s dolphins, 2 common bottlenose dolphins, 1 common dolphin and 1 pilot whale) were defrosted (see Supplementary File S1 for all cetacean samples). A commercial blocking ELISA (INgezim *Brucella* Compac, INGENASA, Madrid, Spain) was used following the manufacturer’s instructions. This competitive ELISA test (*Brucella* cELISA) is a multispecies kit developed for detection of blocking antibodies against LPS of *Brucella* abortus in domestic ruminants and swine serum samples, using a peroxidase-conjugated secondary monoclonal antibody directed to the LPS of *B. abortus*. The dilution of the serum sample for cetaceans is not established for the test and, therefore, sera were diluted as recommended by the manufacturer for ovine and caprine sera (1:5), bovine and porcine sera (1:10), and additionally at 1:20, 1:40, 1:80 and 1:160. Serum of culture-confirmed *Brucella*-infected cases (n = 8) were further diluted to 1:320, 1:640, 1:1280, 1:2560, 1:5120 and 1:10240. Optical density (OD) values were measured at 450 nm within 5 min after the addition of stop solution using a spectrophotometer (FLx800, Bio-Tek Instruments, Winooski, VT, USA). OD values were used to calculate the final results, read as a percentage of inhibition (PI) in comparison to positive and negative control sera included in the kit, with the formula PI = 100 × [1 − (OD test sample/OD negative control)], where OD = optical density. Following the procedures of the test, a sample was classified as positive if the PI in the well was ≥40%.

The possible association between the level of hemolysis and the *Brucella* cELISA result was investigated in a non-published preliminary study. Briefly, the level of hemolysis was visually graded in the first dilution of samples when dispensed onto the ELISA plate into group 1 (non-hemolyzed or slightly hemolyzed serum) and group 2 (hemolyzed serum or hemolyzed blood). A comparison of positive and negative results in *Brucella* cELISA (for 1:20 dilutions and 1:40 dilutions) with the hemolysis score was performed with the Wilcoxon two-sample test (Epi Info Package, www.cdc.gov/epiinfo/index.html (accessed on 19 August 2024)).

### 2.3. Brucella Isolation

*Brucella* isolation, the gold standard for brucellosis diagnosis, was attempted in dolphins with compatible lesions, a positive reaction to Rose Bengal Test or with CeMV infection. Samples used for *Brucella* isolation were frozen swabs from the lateral cerebral ventricle, periventricular cerebral tissue, cerebrospinal fluid (CSF), spleen, mesenteric lymph node, or swabs from spondylytic lesions. Bacterial culture was performed as previously described [8]. Briefly, tissue samples were superficially sterilized, homogenized in saline buffer and cultured in plates of both Farrell and CITA selective media. Isolates were identified as marine *Brucella* using a Bruce-ladder PCR [16]. Confirmation of *Brucella ceti* as the isolated species was conducted by a multiplex PCR adapted from Bruce-ladder [16].

### 2.4. Age Determination

The age of the 57 striped dolphins with serological analysis was estimated using a Gompertz formula established for this species in the Northwestern Mediterranean Sea [17]. Subsequently, animals were divided into four age ranges (fetus, calves, juveniles and adults), considering adulthood as being above 6 years [18].

### 2.5. Data Analysis

Differences between antibody titers in striped dolphins, grouped by their cause of death and age, were compared in two-way contingency tables and using Fisher’s test. R software (version 4.4.0) was used to introduce data and elaborate the tables and StatCalc tool, from EpiInfo (version 7.2.6.0), to retrieve significance of the results. Significance was considered with *p*-value < 0.05.

## 3. Results

### 3.1. Pathological Investigation

**Figure 1:**
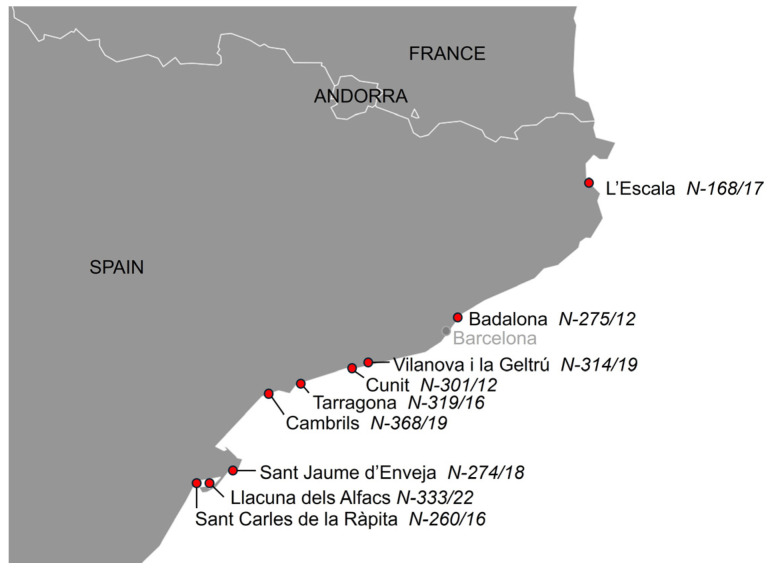
Map showing the stranding location of 9 cetaceans with *Brucella* infection, 7/9 of them with neurobrucellosis.

**Figure 2:**
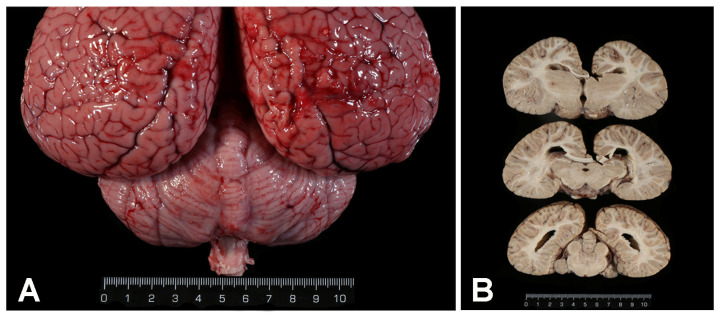
Gross findings in striped dolphins with neurobrucellosis. Scale is in cm. (**A**) Brain, fresh, dorsal view. Cerebellar meninges are turbid. Histologically, there was a lymphoplasmacytic meningitis. Case N-333/22. (**B**) Brain, formalin-fixed. Mild dilation of the ventricular system (hydrocephalus). Case N-314/19.

**Figure 3:**
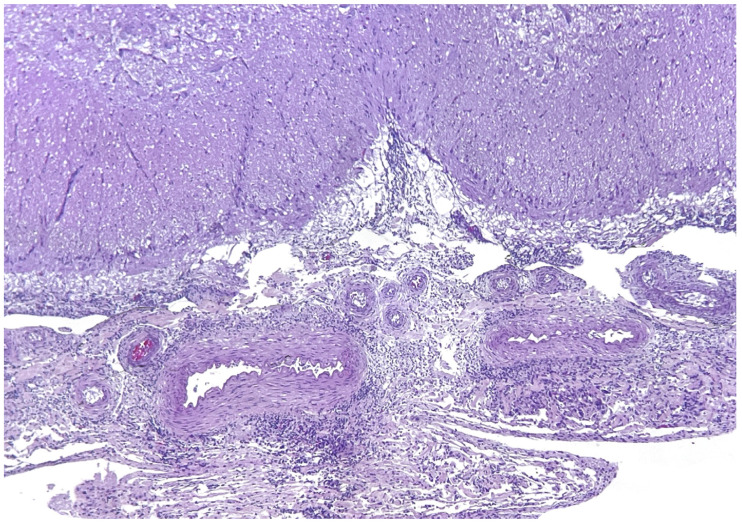
Spinal cord and meninges, hematoxylin and eosin stain. A severe subacute-chronic lymphoplasmacytic meningitis is the hallmark of neurobrucellosis. Case N-333/22.

See Supplementary File S2 for biometrical data, cause of death and ancillary test results in the 123 cetaceans. Nine cases of infection by *Brucella ceti* were detected among the one hundred twenty-three (7.3%) necropsied cetaceans, eight striped dolphins and one common bottlenose dolphin. Seven of the nine dolphins stranded alive and died shortly thereafter (n = 4) or were euthanized due to bad prognosis (n = 3). Five of them showed neurological signs, disorientation or abnormal swimming before death (301/12, 319/16, 368/19, 314/19 and 333/22). Biometric and stranding data from the nine cetaceans with brucellosis are shown in Table 1, and the stranding location is depicted in Figure 1. In two animals, the meningeal turbidity was visible grossly, and in one there was a mild hydrocephalus (Figure 2). Seven striped dolphins had neurobrucellosis, characterized by a severe diffuse or multifocal non-suppurative meningitis or meningoencephalitis, in some cases more intense in the cerebellum and brainstem (Figure 3), and including choroiditis and radiculoneuritis. The other striped dolphin showed a subacute systemic CeMV infection, with an intense encephalitis and less meningeal involvement. Morbilliviral encephalitis was considered the cause of death in this dolphin. The common bottlenose dolphin had ankylosing spondylitis. Macroscopic and microscopic findings of the *Brucella*-infected cases are summarized in Table 2.

### 3.2. Culture of Brucella

*Brucella* culture was attempted in 25 cetaceans, including cases with compatible histological lesions, epididymitis or a positive Rose Bengal test. Three additional dolphins with a positive result in a qPCR against *Brucella* in brain tissue were included. *Brucella ceti* was isolated in 8/25 cetaceans (six striped dolphins with neurobrucellosis, one striped dolphin with subacute morbilliviral encephalitis, and the common bottlenose dolphin with diskospondylitis). In the striped dolphins, *Brucella* was cultured from CNS samples (brain, CSF, ventricle swabs, spinal cord), or mesenteric lymph node, and in the bottlenose dolphin, from the diskospondylitic lesion [8] (see Table 2). The diagnosis of neurobrucellosis in an additional striped dolphin (N-368/19) was based on highly compatible lesions and a low Ct result in the *Brucella* qPCR in CSF. Culture was attempted but the sample was contaminated due to storage problems. A positive *Brucella* PCR result has prompted the inclusion of this case in the neurobrucellosis group for the serological analysis.

The level of haemolysis did not affect the results of the cELISA in our samples from well-preserved cetacean carcasses, as tested in the preliminary study.

### 3.3. Serological Investigation

**Figure 4:**
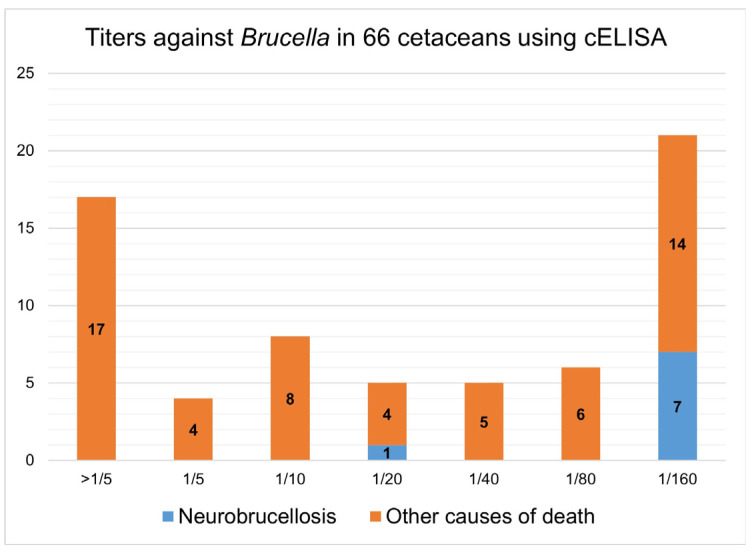
Antibody titers against *Brucella* using a cELISA in 66 cetaceans highlighting *Brucella*-infected cases. Vertical axis shows the absolute number of animals.

A multispecies cELISA was used in an exploratory study to detect antibodies against the LPS of *Brucella* sp. in sixty-six cetaceans, with eight of them infected with *Brucella*. This test has not been validated for cetaceans, and there is no cut-off established to consider one sample as positive or negative. Furthermore, a panel of reference sera of infected and non-infected cetaceans is not available at present. As a consequence, the sensitivity (Se) and specificity (Sp) of the test for cetaceans are unknown. All dolphins were tested at serial dilutions from 1:5 to 1:160, and the last dilution with a PI ≥ 40% was arbitrarily considered the titer of that serum sample, given the fact that the majority of cetaceans (seven of eight) with *Brucella* isolation were positive at the 1:160 dilution. However, a high proportion of sera (21 of 66; 31.8%) were still positive (PI ≥ 40%) at the 1:160 dilution (see Figure 4). The overall *Brucella* antibody estimated prevalence was 33.3% for striped dolphins and 31.8% for all cetaceans using the dilution of 1:160 and the cut-off of PI ≥ 40%. Further serum dilutions performed with *Brucella*-infected cases showed titers of 1:320 (n = 1), 1:640 (n = 2), 1:1280 (n = 2) and 1:5120 (n = 1). The PI for the different dilutions of each cetacean serum is shown in Supplementary File S1.

#### 3.3.1. Serological Results Compared by Cause of Death

Cetaceans investigated serologically are classified by cause of death, species and cELISA results in Table 3. Seropositivity (at dilution 1:160) was only detected in striped dolphins and Risso’s dolphins. Striped dolphins with neurobrucellosis (n = 8) had the highest proportion of seropositives (87.5%). High antibody titers (1:160), however, were also observed in striped dolphins dying from bycatch, CeMV infection, PEM and with cause of death classified as “other”. Animals with neurobrucellosis showed a statistically significant higher proportion of seropositives compared to the groups of bycatch (4/15; 26.6%), striped dolphins not dying from neurobrucellosis (12/49; 24.5%) and the rest of cetaceans (14/66; 21.2%). Regarding other species, only 2/5 Risso’s dolphins were seropositive, a calf dying from maternal separation and a bycaught adult (Table 2). None of the Risso’s dolphins investigated serologically showed lesions compatible with brucellosis, and in the two cases where culture was attempted, the results were negative.

#### 3.3.2. Serology by Age Class

The proportion of seropositive striped dolphins within the different age classes is shown in Table 4. Juveniles, compared to adults, presented a higher proportion of seropositivity (53.9 vs. 29.3%). They comprised the majority of neurobrucellosis cases (25 vs. 4.9%), and this difference was significant with *p* < 0.019. Seropositivity was 33% for both male and female striped dolphins.

#### 3.3.3. Brucella Serology in CeMV-Infected Cetaceans

A CeMV outbreak in the Northwestern Mediterranean Sea accounted for the death of 17 cetaceans from 2016 to 2021, including systemic and chronic CNS cases [15] (see Table 5). The diagnosis was made with histopathology, a positive PCR and/or IHC result in the target organs. High titers against *Brucella* within this group were frequent in dolphins diagnosed with CeMV-chronic CNS forms. Only one of four CeMV cases, where *Brucella* isolation was attempted, yielded a positive result (N-319/16, stranded in 2016, with CNS lesions attributed to CeMV). In four of these cases, *Brucella* culture was attempted and was negative.

## 4. Discussion

The results show that brucellosis is regularly detected in small cetaceans in the Northwestern Mediterranean Sea, and it is the most significant single bacterial cause of death in striped dolphins in the area, confirming previous similar findings from Mediterranean regions [12,15]. *B. ceti* infection and disease was diagnosed in nine out of one hundred twenty-three (7.3%) necropsied cetaceans, with this figure being the first estimated prevalence of the disease in dolphins from any Mediterranean coastal region. In eight striped dolphins, neurobrucellosis was deemed the primary cause of death, whereas *B. ceti* was considered a comorbidity in one striped dolphin with systemic CeMV infection and in a common bottlenose dolphin with *B. ceti* diskospondylitis and a CNS mucormycosis [8].

Understanding the pathogenesis of cetacean neurobrucellosis remains elusive, mostly due to the lack of knowledge about the types of immune responses elicited by *B. ceti* in healthy and in sick cetaceans. It is generally accepted that Th1 responses are relevant in controlling replication of intracellular bacteria. A strong humoral (Th2-mediated) response seems to be unprotective, with antibodies being a more useful indicator of exposure and infection than a proper defense mechanisms (for review, see [19,20]). Accordingly, a study with *B. melitensis* in mice suggested that antibodies are not decisive in the control of infection [21], although antibodies against LPS have proven to confer a certain protection in *B. abortus* [19,22]. As in other mammals, it may be hypothesized that cetaceans with a predominant Th1 response to *B. ceti* survive the infection, and a shift to a Th2 response constitutes the hallmark of disease progression. However, to our knowledge, the specific T cell response against *Brucella* sp. has never been measured in dolphins, and the role of serum antibodies against the bacteria is unknown. Several serologic tests have been used in cetacean species, either developed for terrestrial mammals or adapted to marine mammals [11,23,24,25,26,27,28,29,30]. These studies have shown a high seroprevalence of *Brucella*-infection, ranging from 7.6% to 60%, both in cetaceans and pinnipeds, in many places of the world. However, the Se and Sp of tests designed for terrestrial mammals have not been established for cetaceans, and this knowledge is hindered by the lack of reference panel sera for cetaceans. Bearing in mind these limitations, we applied a commercial cELISA for *Brucella* diagnosis in cetaceans, not to establish Se and Sp values for this test, but rather to better define prevalence of *B. ceti* infection and disease, and to understand the practical utility of serologic results when applied to stranded cetaceans.

We observed a higher seropositivity in striped dolphins with neurobrucellosis than in the total of analyzed cetaceans and in striped dolphins dying from other causes, considering seropositivity when PI ≥ 40% at a serum dilution of 1:160. The causes of death in seropositive animals, however, were varied and included infectious and non-infectious causes, indicating that high antibody titers are not a synonym of active, pathology-related *Brucella* infection. If these cetaceans with high titers represent subclinical forms of latent *Brucella* infections, inducing humoral immunity remains to be studied. Antibodies in humans with brucellosis can persist several months after the remission of symptoms, clinical relapse [31,32] and, in areas with endemicity, repeated infections [31]. In rats inoculated with *B. abortus* antigens, antibodies are present a minimum of 120 days [33]. There is a report of a captive bottlenose dolphin with *Brucella* osteomyelitis with sustained high antibody titers against *Brucella* [34]. Considering this, it is possible that antibodies would be indicative of chronicity rather than accountable for disease development. In humans, whose pathogenesis of neurobrucellosis is often compared to cetaceans, neurobrucellosis is not the most common manifestation of disease [31,32] and occurs in subacute to chronic infections [35,36]. Thus, it is likely that the diagnosed cases presented in this work represent just the tip of the iceberg of an endemicity of *Brucella* infection in the Mediterranean, as suggested previously [15] and supported by the high seroprevalence reported in other studies around the world (see [4] for review).

The variable specificity of serological tests is often attributed to cross-reactions with the other LPS of Gram-negative bacteria. In terrestrial animals, *Campylobacter* spp., *Salmonella* spp., *Pasteurella* spp., *Yersinia enterocolytica* [11], *Francisella tularensis* and *Vibrio cholerae* [37] have been associated with cross-reactions. *Salmonella* spp. have been isolated from free-ranging cetaceans [38], but other terrestrial bacteria are not always found in marine environments. In the most common Gram-negative bacteria associated with marine mammals, the pathogenicity and roles in cross-reaction is unclear. In the seropositive dolphins of this paper, *Psychrobacter phenylpyruvicus* (N-28/21), *Photobacterium damselae* (N-557/17, N-232/18, N-42/18), *Pseudomonas* spp. (N-557/17), *Vibrio* sp. (N-232/18, N-329/18) and *Escherichia coli* (N-329/18) have been isolated.

The presence of high seropositivity in the cases of neurobrucellosis probably indicates that the humoral response is not effective against the pathogen. In our cases, Th1 response cytokines (IL-1 and TNFα) have been detected in *Brucella*-induced meningitis by immunohistochemistry, which could suggest a role of this type of response in brucellosis [39]. In these cases, for unknown reasons, cellular response is ineffective, as studied in other species [40]. Polymorphisms in some molecules of the immune system may also play a role in individual susceptibility [41,42].

Interestingly, the authors observed that juvenile striped dolphins were the age group with higher seroprevalence and proportion of neurobrucellosis. In our cases, this may be due to a larger contribution of anthropogenic interactions as cause of death for adults (21/61; 34.4%), diminishing the relative importance of neurobrucellosis. Immune immaturity as a predisposing cause for neurologic form of brucellosis in juveniles, however, cannot be discarded. Another hypothesis is the occurrence of vertical transmission, with consequent latency of *Brucella* in the tissues and reactivation when new stressors appear for the weaned animal. The results from this work contrast with some serologic studies in human patients, where they found a positive correlation between the age and seroprevalence of *Brucella* antibodies [43,44]. However, this may be due to adults being more exposed in work environment. In Italy and Costa Rica, similar results have been reported, with 6/8 striped dolphins with brucellosis being juveniles in Italy [12] and 28/51 in Costa Rica [45].

A cluster of *Brucella*-seropositive dolphins occurred in the period 2018–2021 in CNS-localized forms of CeMV (n = 5) (see Supplementary File S1). The authors speculate that the previous immune suppression of the systemic phase prompted the increased replication and transmission of subclinical *Brucella*, without causing death, and the persistence of *Brucella* antibodies until the development of the chronic form of CeMV, when the immunosuppressive phase had resolved. Another hypothesis is that the acute phase transiently limited antibody production. At least in the present cases, there is no direct correlation between the massive immunosuppression in acute CeMV infection and the development of neurobrucellosis. In other publications, the two coinfections are more frequent, with 4/8 striped dolphins testing positive for both pathogens [12], although in others there was no correlation [46].

## 5. Conclusions

A seroprevalence of 31.8% against *Brucella* LPS antigens in cetaceans from the Catalan coast of the Mediterranean has been detected. This percentage is significantly higher in cetaceans with neurobrucellosis (87.5%), but there are also high titers in animals with other causes of death. Seropositive species include striped dolphins and Risso’s dolphins. The prevalence of disease associated with *Brucella ceti* was 7.3%.

The cELISA designed for ruminants and pigs may be a potentially sensitive method in the serum samples of cetaceans to predict neurobrucellosis, but the specificity is probably low. This may reflect a proportion of subclinical carriers of the bacteria or the occurrence of cross-reactions. Cetaceans with chronic forms of CeMV seem to have higher antibody titers against *Brucella*. CeMV infection, in the acute immunosuppressive form, may enhance *Brucella* replication in carrier individuals and increase the horizontal transmission of the bacteria. More efforts are needed to dilucidate the epidemiology of the infection and the latency of bacteria in target organs. Research must continue to better understand this disease epidemiology and pathogenesis, a significant cause of mortality in our coast with the potential to alter the population dynamics of these protected species.

## Figures and Tables

**Table 1:** Biometric data and stranding information of the 9 dolphins with brucellosis. J = juvenile. Ad = adult. D = found dead. A = stranded alive. All animals are striped dolphins except N-275/12, which is a common bottlenose dolphin.

ID	Date	Place of Stranding	Age Class	Age Estimation	Length (cm)	Weight (kg)	Sex	A/D
**N-275/12**	23 May 2012	Badalona	Ad	-	300	Unknown	M	D
**N-301/12**	3 June 2012	Cunit	Ad	6.25	184	54.5	F	A
**N-260/16**	19 June 2016	Sant Carles de la Ràpita	J	3.88	170	39.5	F	D
**N-319/16**	8 September 2016	Tarragona	J	5.35	180	47	F	A
**N-168/17**	18 April 2017	L’Escala	Ad	10.39	193	79	M	A
**N-274/18**	24 June 2018	Sant Jaume d’Enveja	J	2.43	152	38	M	A
**N-314/19**	1 September 2019	Vilanova i la Geltrú	J	2.8	159	41	F	A
**N-368/19**	12 October 2019	Cambrils	J	5.23	175	47	M	A
**N-333/22**	22 September 2022	Llacuna dels Alfacs	Ad	6.15	180	47.5	M	A

**Table 3:** Antibody titers in the cELISA against *Brucella* LPS in serum samples of 66 cetaceans. The column on the right (%Pos) shows the percentage of seropositives (PI ≥ 40% at dilution 1:160) in each group. * In one dolphin, the cause of death was encephalitis by CeMV. ^♦^ Chronic forms of CeMV stranded in the period 2018–2021. Sc = *Stenella coeruleoalba*; Gg = *Grampus griseus*; Tt = *Tursiops truncatus*; Dd = *Delphinus delphis*; Gm = *Globicephala melas*.

Species	Cause of Death	n	Antibody Titer							
			>1:5	1:5	1:10	1:20	1:40	1:80	1:160	%Pos
*Sc*	Neurobrucellosis *	8				1			7	87.5%
	Bycatch	15	4	1	3		1	2	4	26.6%
	CeMV	12	5	1			1	1	4 ^♦^	33.3%
	Infectious/inflammatory	9	1	1	2	3		2		0%
	PEM	4		1	1				2	50%
	Mother–calf separation	2	1		1					0%
	Other	4	1			1			2	50%
	Unknown	3	1				2			0%
	TOTAL	57	13	4	7	5	4	5	19	33.3%
*Gg*	Sinusitis *Crassicauda*	3	2					1		0%
	Bycatch	1							1	100%
	Mother–calf separation	1							1	100%
	TOTAL	5	2					1	2	40%
*Tt*	Mother–calf separation	1	1							0%
	Infectious/inflammatory	1			1					0%
	TOTAL	2	1		1					0%
*Dd*	Unknown	1					1			0%
*Gm*	CeMV	1	1							0%
TOTAL		66	45						21	31.8%

**Table 4:** Seropositivity for *Brucella* (left column) and neurobrucellosis cases (right column) by age class. Serum was available in n = 57 striped dolphins. Right column shows the proportion of neurobrucellosis as cause of death in all striped dolphins, with or without serological analysis.

	Seropositives (%)	Neurobrucellosis (%)
Adults	12/41 (29.3%)	3/61 (4.9%)
Juveniles	7/13 (53.9%)	5/20 (25%)
Calves	0/3 (0%)	0/13 (0%)
Fetus	0	0/1 (0%)
Total	19/57 (33.3%)	8/97 (8.2%)

**Table 5:** Antibody titers against *Brucella* spp. in cetaceans infected with CeMV. * *Brucella* coinfection. Sc = *Stenella coeruleoalba*; Gm = *Globicephala melas*; J = juvenile; Ad = Adult.

ID	Species	Age Class	CeMV Form	Titer
N-319/16	Sc	J	Systemic *	1/160
N-044/17	Sc	J	Systemic	>1:5
N-045/17	Sc	J	Systemic	>1:5
N-077/17	Sc	J	Systemic	>1:5
N-454/17	Sc	J	Systemic	>1:5
N-488/17	Sc	Ad	Systemic	1/5
N-497/17	Sc	Ad	Systemic	1/10
N-604/17	Sc	Ad	Chronic	>1:5
N-001/18	Sc	Ad	Chronic	1/40
N-232/18	Sc	J	Chronic	1/160
N-293/18	Sc	Ad	Chronic	1/160
N-362/18	Sc	Ad	Chronic	1/160
N-361/19	Sc	J	Chronic	1/160
N-334/21	Sc	Ad	Chronic	1/80
N-023/21	Gm	J	Chronic	>1:5

**Table 2:** Summary of the signalment and pathological findings in 8 striped dolphins and a bottlenose dolphin (N-275/12) (*, previously published in [8]) diagnosed with brucellosis or with *Brucella* infection. CSF = cerebrospinal fluid. Only lesions attributable to *Brucella ceti* or relevant to cause of death are included.

ID	Macroscopic Lesions	Histopathology	*Brucella* Culture	Others	Cause of Death	Available Serum
N-275/12 *	Chronic suppurative diskospondylitis Multifocal brain malacia	Mycotic pyogranulomatous-necrotizing meningoencephalomyelitis	**Positive** (vertebral abscess)	Mycotic encephalitis	Mycotic encephalitis	No
N-301/12 *	Not relevant	Non-suppurative meningoencephalitis, diffuse, more intense in cerebellum, brainstem and spinal cord; choroiditis; radiculoneuritis	**Positive** (brain)	-	Neurobrucellosis	Yes
**N-260/16**	Emaciation; cerebral edema; absence of recent ingesta	Non-suppurative meningoencephalitis, diffuse, more intense in brainstem; choroiditis; radiculoneuritis, multifocal and milder	**Positive** (brain)	-	Neurobrucellosis	Yes
**N-319/16**	Low body condition; absence of recent ingesta; meningeal congestion	Non-suppurative meningoencephalitis	**Positive** (brain, ventricle swab)	CeMV intensely positive (RT-PCR and IHC)	CeMV encephalitis	Yes
**N-168/17**	Absence of recent ingesta; meningeal and CSF turbidity	Non-suppurative meningitis, more intense in brainstem and cerebellum	**Positive** (lateral ventricle swab, spinal cord, mesenteric LN, brain, CSF)	-	Neurobrucellosis	Yes
**N-274/18**	Loss of body condition; brain edema and meningeal hemorrhage	Non-suppurative meningitis, diffuse, with multifocal encephalitis; choroiditis	**Positive** (lateral ventricle swab)	*Aeromonas sobria* and *P. damselae* in lung, liver, CNS	Neurobrucellosis	Yes
**N-314/19**	CSF turbidity; brain edema and meningeal hemorrhage; mild hydrocephalus	Non-suppurative meningoencephalitis, more intense in brainstem, cerebellum and thalamus; choroiditis; radiculoneuritis, multifocal and milder	**Positive** (CSF)	-	Neurobrucellosis	Yes
**N-368/19**	Emaciation; absence of recent ingesta; CSF turbidity	Non-suppurative meningitis, localized in brainstem and, with less intensity, cerebellum	Negative (contaminated sample) **PCR intensely positive** in CSF.	-	Neurobrucellosis	Yes
**N-333/22**	Slight loss of body condition; absence of regent ingesta; brain edema; meningeal and CSF turbidity	Non-suppurative meningitis, localized in brainstem and cerebellum, choroiditis	**Positive** (brain, CSF)	-	Neurobrucellosis	Yes

## Data Availability

Data supporting reported results can be found in Supplementary Materials.

## Supplementary Materials

The following supporting information can be downloaded at https://www.mdpi.com/article/10.3390/ani14162417/s1, File S1: Percentage of inhibition of all cELISA tested cetaceans; File S2: Biometric data, ancillary tests and cause of death of the 131 studied cetaceans.
